# Automated oxygen control for very preterm infants and neurodevelopmental outcome at 2 years−a retrospective cohort study

**DOI:** 10.1007/s00431-023-04809-4

**Published:** 2023-01-25

**Authors:** Hylke H. Salverda, N.Nathalie J. Oldenburger, Monique Rijken, R.Ratna N. G. B. Tan, Arjan B. te Pas, Jeanine M. M. van Klink

**Affiliations:** 1 Department of Paediatrics, Division of Neonatology, Willem-Alexander Children’s Hospital, Leiden University Medical Center, PO Box 9600, Leiden, the Netherlands; 2 Department of Paediatrics, Division of Psychology, Willem-Alexander Children’s Hospital, Leiden University Medical Center, Leiden, the Netherlands

**Keywords:** Hypoxemia, Hyperoxia, Closed-loop, Algorithm, Neonate, Respiratory, Follow-up

## Abstract

Faster resolution of hypoxaemic or hyperoxaemic events in preterm infants may reduce long-term neurodevelopmental impairment. Automatic titration of inspiratory oxygen increases time within the oxygen saturation target range and may provide a more prompt response to hypoxic and hyperoxic events. We assessed routinely performed follow-up at 2 years of age after the implementation of automated oxygen control (AOC) as standard care and compared this with a historical cohort. Neurodevelopmental outcomes at 2 years of age were compared for infants born at 24–29 weeks gestational age before (2012–2015) and after (2015–2018) the implementation of AOC as standard of care. The primary outcome was a composite outcome of either mortality or severe neurodevelopmental impairment (NDI), and other outcomes assessed were mild-moderate NDI, Bayley-III composite scores, cerebral palsy GMFCS, and CBCL problem behaviour scores. A total of 289 infants were included in the pre-AOC epoch and 292 in the post-AOC epoch. Baseline characteristics were not significantly different. Fifty-one infants were lost to follow-up (pre-AOC 6.9% (20/289), post-implementation 10.6% (31/292). The composite outcome of mortality or severe NDI was observed in 17.9% pre-AOC (41/229) vs. 24.0% (47/196) post-AOC (*p* = 0.12). No significant differences were found for the secondary outcomes such as mild-moderate NDI, Bayley-III composite scores, cerebral palsy GMFCS, and problem behaviour scores, with the exception of parent-reported readmissions until the moment of follow-up which was less frequent post-AOC than pre-AOC.

*Conclusion*: In this cohort study, the implementation of automated oxygen control in our NICU as standard of care for preterm infants led to no statistically significant difference in neurodevelopmental outcome at 2 years of age.

**Table:** 

**What is Known:** *• Neurodevelopmental outcome is linked to hypoxemia, hyperoxaemia and choice of SpO2 target range.* *• Automated titration of inspired oxygen may provide a faster resolution of hypoxaemic and hyperoxaemic events.*	
**What is New:** * • This cohort study did not find a significant difference in neurodevelopmental outcome at two years of age after implementing automated oxygen control as standard of care.*

**Supplementary Information:**

The online version contains supplementary material available at 10.1007/s00431-023-04809-4.

## Introduction

Maintaining appropriate oxygenation in preterm infants admitted to the neonatal intensive care unit (NICU) has proved challenging but of importance to outcome. Neonatal morbidity and mortality are linked to hypoxemia, hyperoxaemia, and choice of SpO_2_ target range [1–3]. Post hoc analysis of the Canadian Oxygen Trial data associated adverse neurodevelopmental outcome with hypoxia, in particular hypoxic episodes lasting more than 1 min [4]. Hunt et al. reported similar evidence in their report of home-monitored preterm and term infants: Having five or more apneic/bradycardic events was associated with a 5.6-point lower mental development index [5]. Neither study showed a significant difference for episodes under 1 min. These results could indicate that faster resolution of hypoxaemic or hyperoxaemic events may reduce long-term neurodevelopmental impairment (NDI).

Automatic titration of inspired oxygen (FiO_2_) can provide a more prompt response to these events than when titration is done manually by bedside staff. With the aim of keeping SpO_2_ in a specified target range, an automated oxygen controller (AOC) built into the respirator continually evaluates on the measured SpO_2_ and makes changes in FiO_2_ where necessary. Beside potential benefits to workload, it has been demonstrated that several commercially available controllers increase the time preterm infants spent within the target range while used for 2–24 h periods, and prolonged episodes of hypoxemia and hyperoxaemia are reduced [6–13]. This was also reflected in a study [14] done in our centre while using an AOC as standard of care. However, to date, evidence on clinically relevant neonatal outcome is scarce [15] and lacking altogether beyond the neonatal period.

In August 2015, AOC was implemented as standard of care in the NICU of the Leiden University Medical Center (LUMC). Recently, we reported the effect of this implementation on the clinical outcome of preterm infants during admission [15]. Implementation did not lead to a change in mortality or rate of retinopathy of prematurity (ROP), necrotising enterocolitis (NEC), intraventricular haemorrhage (IVH), periventricular leukomalacia (PVL), or bronchopulmonary dysplasia (BPD), but there was less invasive ventilation in the post-implementation cohort. Thus far, none of the studies comparing manual oxygen control with AOC have reported the effect on long-term neurodevelopmental outcome. We therefore aimed to compare the neurodevelopmental outcome of preterm infants born before and after the implementation of AOC as standard care with routinely performed 2-year follow-up assessment.

## Materials and methods

### Study design

A retrospective study was conducted in the NICU of the LUMC, a tertiary-level perinatal centre with annually around 100 intensive care admissions of infants born before 30 weeks of gestation. A statement of no objection (G19.075) for obtaining and publishing the anonymised data was provided by the ethical board of the LUMC.

Infants admitted to the NICU between May 1, 2012, and December 31, 2018, and born between 24 and 29 weeks and 6 days of gestation were included in the analysis. Infants were excluded from the study if they were admitted > 24 h after birth, required no invasive or non-invasive respiratory support during their admission, or had major congenital abnormalities.

The pre-implementation cohort included patients admitted between May 1, 2012, and June 17, 2015, that received FiO_2_ manually titrated by bedside staff according to local guidelines. The post-implementation cohort consisted of infants admitted from October 18, 2015, to December 2018, taking into consideration a washout period of 4 months.

### Data collection

All data were obtained from our patient data management systems (Metavision; IMDsoft, Tel Aviv, Israel and HiX; ChipSoft, Amsterdam, The Netherlands). Infants were invited for follow-up at 24 months corrected age for assessment by a neonatologist, paediatric physiotherapist, and paediatric psychologist. Respectively, they were responsible for the general and neurological examination, the assessment of motor function, and the assessment of cognitive functioning. The primary outcome was a composite outcome of mortality or severe NDI. Secondary outcomes were as follows: mild to moderate NDI, early mortality (mortality until 1 month after corrected term age), late mortality (mortality between 1 month after corrected term age and 2-year follow-up), motor and cognitive development scores, visual impairment, hearing loss, cerebral palsy, behavioural functioning, and number of readmissions. Severe NDI was defined as at least one of the following: cerebral palsy (Gross Motor Function Classification System (GMFCS) [16] ≥ level 3), Bayley-III-NL cognitive or motor scores less than two standard deviations under the mean, severe bilateral visual impairment or blindness, and/or bilateral sensorineural hearing loss or deafness needing hearing aids or cochlear implants. Mild to moderate NDI was defined as cerebral palsy GMFCS level 1 or 2, Bayley-III-NL cognitive or motor scores below one standard deviation under the mean, and mild visual and/or hearing impairment. Motor and cognitive development were assessed using the Bayley Scales of Infant and Toddler Development Third Edition, Dutch version (Bayley-III-NL) [17, 18]. As the minimum cognitive composite score using the Bayley III is 55, children failing to achieve this were nominally assigned a score of 54. Bayley III floor motor composite score is 46; hence, children failing to achieve this were nominally assigned a score of 45 [19]. Visual impairment was classified as mild (needing treatment by an ophthalmologist or orthoptist), impaired vision with the ability to see, or blind. Hearing loss was defined as mild, neurosensory hearing loss, or deaf*.* Behavioural outcome was assessed using the Child Behavioural Checklist (CBCL 1.5–5 years) completed by parents [20]. A classification of level 3 or higher on the GFMCS was considered severe [16]. When data could not be collected from the patient data management systems, the Dutch Perinatal and Neonatal register (Landelijke Neonatale Registratie) or other follow-up centres (university hospitals, revalidation centres) were consulted to complement the data.

### Oxygen titration

During almost the entire study period, the AVEA ventilator (Vyaire, Yorba Linda CA, USA) was used for respiratory support; after August 2015, this involved AOC by the CLiO_2_ (Closed Loop of inspired Oxygen) controller [8]. As of November 2018, newly born preterm infants were supported by the SLE6000 ventilator (SLE, London, UK) with the OxyGenie option for AOC [21]. Following recent European guidelines [22], in November 2014, we changed the SpO_2_ target range in our NICU from 85–95% to 90–95%.

### Data analysis

Data are reported as mean (SD), median (range), or number (percentage) as appropriate, with standard tests for normality. Statistical comparison was executed using an independent *t*-test, a Mann–Whitney *U* test, and a chi-square or Fisher’s exact test as appropriate. Statistical analyses were performed using IBM SPSS Statistics for Windows, version 25 (IBM, Armonk, New York, USA). Two-sided *P* values of < 0.05 were considered statistically significant.

## Results

### Patient characteristics and neonatal period

In the period of 2012–2018, 588 infants were born in the LUMC or transferred to the NICU within 24 h after birth, 293 infants in the pre-implementation period (pre-AOC) versus 295 infants in the post-implementation cohort (post-AOC). There were no differences in baseline characteristics (Table 1). In the neonatal period, there were similar rates of IVH, PVL, and laser coagulation for ROP. More details on the neonatal period can be found in our previous study [15]. Three children were excluded from analysis due to major congenital abnormalities affecting neurodevelopment (2 pre-AOC, 1 post-AOC), and 4 were excluded because they moved to a different country (2 pre-AOC, 2 post-AOC), leaving 289 infants in the pre-AOC group and 292 infants in the post-AOC group. We were unable to obtain any follow-up data for 51 infants (pre-AOC 6.9% (20/289), post-implementation 10.6% (31/292), baseline characteristics Supplemental (Table 1–2).

**Table 1:** Patient characteristics and neonatal outcome

Patient characteristics (*N* = 581)	Pre-AOC (*N* = 289)	Post-AOC (*N* = 292)	*P* value^a^
Gestational age in weeks^days^, median [IQR]	28^2^ [26^6^–29^0^]	28^0^ [26^3^–28^6^]	0.09
Birth weight in grams, mean (SD)	1037 (291)	1037 (261)	1.00
Males, *n* (%)	162 (56.1)	155 (53.1)	0.47
Antenatal corticosteroids, *n* (%)	246 (86.0)	253 (87.5)	0.59
Caesarean delivery, *n* (%)	143 (49.5)	154 (52.7)	0.43
Multiple pregnancy, *n* (%)	115 (39.8)	97 (33.1)	0.10
of which monochorionic twins, *n* (%)	71 (61.7)	62 (63.9)	0.36
Perinatal asphyxia, *n* (%)	4 (1.4)	8 (2.7)	0.25
Apgar score at 5 min, median (range)	8 (2–10)	8 (1–10)	0.90
Intraventricular haemorrhage (≥ stage 2), *n* (%)	55 (19.0)	49 (16.8)	0.48
Periventricular leukomalacia (≥ stage 2), *n* (%)	4 (1.4)	6 (2.1)	0.53
Received laser coagulation for ROP, *n* (%)	13 (5.3)	14 (5.6)	0.85
Bronchopulmonary dysplasia			
Severe, *n* (%)	36 (14.2)	48 (18.8)	
Moderate, *n* (%)	12 (4.7)	4 (1.6)	0.10
Mild, *n* (%)	44 (17.3)	37 (14.5)	

*AOC* automated oxygen control, *ROP* retinopathy of prematurity

^a^Statistical analysis with independent *T*-test, *χ*^2^, or nonparametric Mann–Whitney *U* test as appropriate

**Table 2:** Outcomes at 2 years follow-up

	Pre-AOC	Post-AOC	*P* value^a^
Adverse outcome (severe NDI or death)	41 (17.9)	47 (24.0)	0.12
Severe NDI, *n* (%)	10 (5.1)	13 (8.0)	0.25
Early death, *n* (%)	30 (10.2)	32 (10.8)	0.82
Late death, *n* (%)	1 (0.4)	2 (0.8)	1.00
Mild-moderate NDI, n (%)	82 (38.5)	78 (43.8)	0.29
BSID III composite motor score, median [IQR]	97 [89 – 107]	98 [89–109]	0.18
Score 70–85, *n* (%)	25 (12.6)	25 (12.8)	0.94
Score < 70, *n* (%)	5 (2.5)	11 (5.6)	0.12
BSID III composite cognitive score, median [IQR]	96 [87–101]	96 [91–105]	0.23
Score 70–85, *n* (%)	31 (13.4)	26 (12.7)	0.85
Score < 70, *n* (%)	7 (3.0)	7 (3.4)	0.81
CBCL externalising *T* score, mean (SD)	51 (10)	50 (10)	0.69
CBCL internalising *T* score, median [IQR]	47 [41–55]	47 [41–55]	0.59
Visual impairment, *n* (%)			
None	213 (90.6)	166 (86.0)	0.25
Mild impairment	19 (8.1)	25 (13.0)	
Limited vision	3 (1.3)	2 (1.0)	
Blind	0 (0)	0 (0)	
Hearing loss, *n* (%)			
None	229 (98.3)	185 (96.4)	
Mild hearing loss	2 (0.9)	3 (1.6)	0.23
Abnormal, neurosensory hearing loss	0 (0)	3 (1.6)	
Deaf	2 (0.9)	1 (0.5)	
Cerebral palsy GMFCS, *n* (%)			
None	200 (84.7)	190 (88.0)	0.73
Level 1	30 (12.7)	21 (9.7)	
Level 2	4 (1.7)	4 (1.9)	
Level 3	2 (0.8)	1 (0.5)	
Level 4	0 (0)	0 (0)	
Level 5	0 (0)	0 (0)	
Readmissions until follow-up, *n* (%)			
None	130 (57.0)	149 (69.3)	
1–3	83 (36.4)	50 (23.3)	0.01
> 3	15 (6.6)	17 (7.4)	

*AOC* automated oxygen control, *NDI* neurodevelopmental impairment, *BSID* Bayley Scales of Infant and Toddler Development, *CBCL* Child Behaviour Checklist, *GMFCS* Gross Motor Function Classification System

^a^Statistical analysis with independent *T*-test, *χ*^2^, Fishers’ exact, or nonparametric Mann–Whitney *U* test as appropriate

### Outcomes at 2-year follow-up

The composite primary outcome of mortality or severe NDI occurred in 17.9% (41/229; Table 2) before the implementation of AOC versus 24.0% (47/196) after implementation (*p* = 0.12). The majority was related to neonatal death (10.2% (30/289) vs. 10.8% (32/292) *p* = 0.82). After the neonatal period 0.4% (1/259) vs. 0.8% (2/259; *p* = 0.56), children died. Severe NDI was observed in 5.1% (10/198) before vs. 8.0% (13/162) after the implementation of AOC (*p* = 0.25). Mild to moderate NDI was detected in 38.5% (82/213) infants pre-AOC vs. 43.8% (78/178) after implementation (*p* = 0.29).

The median Bayley-III composite motor score was 97 (89–107) pre-AOC and 98 (89–109) post-AOC (*p* = 0.18). The composite cognitive score was 96 (87–101) vs. 96 (91–105) (*p* = 0.23). No significant differences were found in rates of scores between 1 and 2 SD under the mean (score 70–85) and below − 2 SD under the mean (score < 70), for motor nor cognitive. Motor scores, respectively, for pre- vs. post-implementation cohorts: 12.6% (25/199) of the children had scores between 1 and 2 SD under the mean and 2.5% (5/199) below − 2 SD vs. 12.8% (25/195, *p* = 0.94) and 5.6% (11/195, *p* = 0.12) after implementation. Cognitive scores in the pre-implementation group were found in 13.9% (31/232) between 1 and 2 SD under the mean and under 2 SD under the mean in 3.0% (7/232) vs. 12.7% (26/204, *p* = 0.85) and 3.4% (7/204, *p* = 0.81), respectively. We found no significant differences between groups in externalising (51 ± 10 vs. 50 ± 10, *p* = 0.69) or internalising (47 [41–55] vs. 47 [41–55], *p* = 0.59) problem behaviour scores.

Neurological examination, cerebral palsy GMFCS scores, visual impairment, and hearing loss yielded no significant differences. However, parents did report significantly fewer readmissions until the moment of follow-up in the post-AOC group (*p* = 0.002).

## Discussion

This is the first study to report data on long-term neurodevelopmental follow-up at 2 years corrected age in very preterm infants treated with automated oxygen titration as standard of care compared with manual oxygen titration as standard of care. Implementation of automated oxygen titration did not lead to a change in mortality or neurodevelopmental outcome at 2 years. Although earlier studies [6–14] demonstrate an increase in time within the target range when using automated oxygen titration, we were not able to demonstrate an effect on neurodevelopmental outcome in this large cohort.

To date, there is little data on clinically relevant outcome of infants receiving automated oxygen titration. There is no data available on neurodevelopmental outcome after usage of AOC, and data on follow-up of preterm infants from non-AOC studies are difficult to compare with, because they involved non-standard interventions [23, 24], infants born almost 15 years ago [25, 26], or had a study population that had markedly different characteristics [27, 28]. The outcomes of both groups in our study are similar to the outcome of a previous cohort study from our centre [26].

The reason for fewer readmissions after the implementation of automated oxygen titration is not apparent from our data. Rates of BPD, ROP, and other morbidity potentially requiring re-hospitalisation are similar, although we did find fewer ventilation days in our previous study for the post-AOC cohort [29].

A failure to demonstrate an impact on neurodevelopmental outcome after implementing automated titration can have several causes. In the previous study on achieved target range time in our NICU, we demonstrated that although infants spent more time within the target range overall, this was mainly attributed to a reduction in time above the target range. In fact, using the CLiO_2_ controller led to a 6% increase of time spent under the SpO_2_ target range (90–95%). This increase was mainly just below the (85–90%) target range while still having a similar proportion of hypoxaemia (< 80%). If indeed more time spent under the target range is where neurodevelopmental improvement can be gained, the lack of improvement in this area could explain the lack of impact on neurodevelopmental outcome. Furthermore, as reported before, it could be that outcome is more largely influenced by the frequency and duration of hypoxia and hypoxic events [30], which were not investigated in our previous study nor in most other automated oxygen controller studies. Also, preterm infants can experience many potentially harmful stimuli and events before being tested at 2 years of corrected age, in particular during the neonatal phase. Oxygenation deviations during respiratory support may play only a minor role in the eventual neurodevelopmental outcome, meaning only very large randomised studies are able to demonstrate a statistically significant difference. Thirdly, neonatal care is a rapidly developing field with frequent changes to standard of care. Some of these unmeasured factors may influence the results in either direction. Finally, some of the adverse outcomes are relatively rare. If the effect of automated oxygen control is modest, a large clinical trial would be needed to observe an effect. Our study has a relatively modest convenience sample of 581 infants. Currently, the FiO2-C trial randomises between automated oxygen control or manual titration during the entire NICU stay and will investigate the effect on clinical and neurodevelopmental outcomes at 24 months of corrected age [29]. As their reported sample size is 2340 participants and because it is a prospective interventional study, it is better equipped to observe an effect. However, all commercially available devices are allowed, which may diminish the effect. The study is projected to run until December 2022.

A change in target range from 85–95% to 90–95% may influence the time spent in (mild) hypoxia. In our case, one would expect that the 76% (223/293) infants in the pre-implementation group born before November 2014 spent more time in the 85–90% range, as the lower limit was changed from 85 to 90%. The achieved proportion of time in the 85–90% range based on 1 min-values of the pre- and post-implementation data show no difference while infants received oxygen (pre-AOC 10.9 [8.6–13.5]%, post-AOC 10.4 [7.7–12.7]%, *p* = 0.09) and a 1.8% difference when considering the entire period of respiratory support (pre-AOC 5.5 [1.7–9.8]%, post-AOC 3.7 [1.6–7.6]%, *p* = 0.002; unpublished data). Van Zanten et al. reported before that the change of lower limit led to a reduction in achieved time within the 80–90% range in our unit, but time spent in hypoxia (SpO_2_ < 80%) was not different [31].

One of the inherent limitations of a retrospective design is the rate of missing data (loss to follow-up in this study: pre-AOC 6.9%, post-AOC 10.6%), which is unfortunately frequently high in follow-up research. The majority of missing children were transferred to another university NICU in the neonatal phase and had subsequent follow-up there; therefore, we expect them to be missing at random and not related to neurodevelopmental outcome. However, children lost to follow-up may be under treatment in a special care facility and therefore not missing at random. Parents may be less inclined to present their child for follow-up when they already receive regular tests in such a facility. To limit biased results due to missing such children, we requested data for all children tested elsewhere. Another strength of the study is that we have a relatively large cohort in which we had few exclusion criteria, meaning the results are generalisable to other NICUs in a similar setting. Furthermore, children are tested by trained professionals as part of a standardized national follow-up programme, improving the repeatability and reliability of the assessment of neurodevelopmental outcome. There may be several confounders in the study which we have not been able to adjust for, such as the type of automated oxygen controller, gestational age, and bronchopulmonary dysplasia. Despite most data being collected prospectively during standard follow-up, minimising recall bias, missing values for one of the outcomes was still relatively common. This precludes us from adjusting for these confounders and is a limitation of this study.

Besides fewer parent-reported readmissions, no change in outcome occurred after the implementation of automated oxygen control. It is reassuring that outcomes did not deteriorate and that outcome of our follow-up is similar to earlier reported data. Our results show no signs children are affected negatively by using an automated oxygen controller, whereas there are benefits for staff workload.

## Conclusion

In this cohort study, the implementation of automated oxygen control in our NICU as standard of care for preterm infants led to no significant difference in neurodevelopmental outcome at 2 years of age.

## Supplementary Information

Below is the link to the electronic supplementary material.

Supplementary file1 (DOCX 19 KB)

## Data Availability

Data are available on reasonable request.

## Abbreviations

AOC: Automated oxygen controller
BPD: Bronchopulmonary dysplasia
BSID-III-NL: Bayley Scales of Infant and Toddler Development-Third Edition – NL
CBCL: Child Behaviour Checklist
FiO_2_: Fraction of inspiratory oxygen
GMFCS: Gross Motor Function Classification System
IVH: Intraventricular haemorrhage
NDI: Neurodevelopmental impairment
NEC: Necrotising enterocolitis
NICU: Neonatal intensive care unit
PVL: Periventricular leukomalacia
ROP: Retinopathy of prematurity
SpO_2_: Oxygen saturation measured by pulse-oximetry
