# Supplementary material for: Automated oxygen control for very preterm infants and neurodevelopmental outcome at 2 years−a retrospective cohort study
Source: Eur J Pediatr. 2023 Jan 25;182(4):1593–9. doi: 10.1007/s00431-023-04809-4 (PMC10167103; doi:10.1007/s00431-023-04809-4)
Supplement: Supplementary file 1 — Supplementary file1 (DOCX 19 KB) [file 431_2023_4809_MOESM1_ESM.docx]

Supplemental material

| **Patient characteristics Pre-AOC** | **Data available**  **N = 269** | **Loss to follow-up**  **N = 20** | **P value*** |
| --- | --- | --- | --- |
| Gestational age in weeks^days^, median [IQR] | 28^2^ [26^5^ – 29^0^] | 28^3^ [27^6^ – 29^1^] | 0.09 |
| Birth weight in grams, mean (SD) | 1033 (284) | 1080 (378) | 0.49 |
| Males, n (%) | 151 (56.1) | 11 (55.0) | 0.92 |
| Antenatal corticosteroids, n (%) | 227 (85.3) | 19 (95.0) | 0.23 |
| Caesarean delivery, n (%) | 128 (47.6) | 15 (75.0) | 0.02 |
| Multiple pregnancy, n (%) | 104 (38.7) | 11 (55.0) | 0.15 |
| of which monochorionic twins, n (%) | 64 (61.5) | 7 (63.6) | 0.29 |
| Perinatal asphyxia, n (%) | 4 (1.5) | 0 (0.0) | 1.00 |
| Apgar score at 5 minutes, median (range) | 8 (2-10) | 8.5 (6-10) | 0.03 |
| Intraventricular haemorrhage (≥ stage 2), n (%) | 54 (20.1) | 1 (5.0) | 0.14 |
| Periventricular leukomalacia (≥ stage 2), n (%) | 3(1.1) | 1 (5.0) | 0.25 |
| Received laser coagulation for ROP, n (%) | 12 (5.2) | 1 (5.9) | 1.00 |
| Bronchopulmonary dysplasia |  |  |  |
| severe, n (%) | 34 (14.4) | 2 (11.1) | 0.29 |
| moderate, n (%) | 12 (5.1) | 0 (0.0) |  |
| mild, n (%) | 43 (18.2) | 1 (5.6) |  |

Table 1: Pre-implementation Automated Oxygen Control loss to follow-up characteristics

AOC, Automated Oxygen Control; ROP, Retinopathy of prematurity;

*Statistical analysis with independent T-test, χ^2^, Fisher’s exact, or nonparametric Mann-Whitney U test as appropriate

Table 2: Post-implementation Automated oxygen control loss to follow-up characteristics

| **Patient characteristics Post-AOC** | **Data available**  **N = 261** | **Loss to follow-up**  **N = 31** | **P value*** |
| --- | --- | --- | --- |
| Gestational age in weeks^days^, median [IQR] | 28^0^ [26^1^ – 28^5^] | 28^6^ [27^5^ – 29^4^] | 0.001 |
| Birth weight in grams, mean (SD) | 1020 (263) | 1175 (193) | 0.002 |
| Males, n (%) | 136 (52.1) | 19 (61.3) | 0.33 |
| Antenatal corticosteroids, n (%) | 225 (87.2) | 28 (90.3) | 0.62 |
| Caesarean delivery, n (%) | 140 (53.6) | 14 (45.2) | 0.37 |
| Multiple pregnancy, n (%) | 83 (31.8) | 14 (45.2) | 0.14 |
| of which monochorionic twins, n (%) | 50 (60.2) | 12 (85.7) | 0.08 |
| Perinatal asphyxia, n (%) | 8 (2.7) | 0 (0.0) | 1.00 |
| Apgar score at 5 minutes, median (range) | 8 (1-10) | 8 (2-10) | 0.36 |
| Intraventricular haemorrhage (≥ stage 2), n (%) | 45 (17.2) | 4 (12.9) | 0.54 |
| Periventricular leukomalacia (≥ stage 2), n (%) | 6 (2.3) | 0 (0.0) | 1.00 |
| Received laser coagulation for ROP, n (%) | 13 (6.0) | 1 (3.3) | 1.00 |
| Bronchopulmonary dysplasia |  |  |  |
| severe, n (%) | 47 (20.9) | 1 (3.3) | 0.051 |
| moderate, n (%) | 4 (1.8) | 0 (0.0) |  |
| mild, n (%) | 34 (15.1) | 3 (10.0) |  |

AOC, Automated Oxygen Control; ROP, Retinopathy of prematurity;

*Statistical analysis with independent T-test, χ^2^, Fisher’s exact or nonparametric Mann-Whitney U test as appropriate
